# An mHealth App for Self-Management of Chronic Lower Back Pain (Limbr): Pilot Study

**DOI:** 10.2196/mhealth.8256

**Published:** 2018-09-17

**Authors:** Aliza Selter, Christina Tsangouri, Sana B Ali, Diana Freed, Adrian Vatchinsky, James Kizer, Arnaud Sahuguet, Deneen Vojta, Vijay Vad, JP Pollak, Deborah Estrin

**Affiliations:** 1 Cornell Tech New York, NY United States; 2 Healthix Inc New York, NY United States; 3 UnitedHealth Group Research & Development Minnetonka, MN United States; 4 Hospital for Special Surgery New York, NY United States

**Keywords:** low back pain, chronic disease, self-assessment, telemedicine, self-management, activities of daily living, pain, rehabilitation

## Abstract

**Background:**

Although mobile health (mHealth) interventions can help improve outcomes among patients with chronic lower back pain (CLBP), many available mHealth apps offer content that is not evidence based. Limbr was designed to enhance self-management of CLBP by packaging self-directed rehabilitation tutorial videos, visual self-report tools, remote health coach support, and activity tracking into a suite of mobile phone apps, including Your Activities of Daily Living, an image-based tool for quantifying pain-related disability.

**Objective:**

The aim is to (1) describe patient engagement with the Limbr program, (2) describe patient-perceived utility of the Limbr program, and (3) assess the validity of the Your Activities of Daily Living module for quantifying functional status among patients with CLBP.

**Methods:**

This was a single-arm trial utilizing a convenience sample of 93 adult patients with discogenic back pain who visited a single physiatrist from January 2016 to February 2017. Eligible patients were enrolled in 3-month physical therapy program and received the Limbr mobile phone app suite for iOS or Android. The program included three daily visual self-reports to assess pain, activity level, and medication/coping mechanisms; rehabilitation video tutorials; passive activity-level measurement; and chat-based health coaching. Patient characteristics, patient engagement, and perceived utility were analyzed descriptively. Associations between participant characteristics and program interaction were analyzed using multiple linear regression. Associations between Your Activities of Daily Living and Oswestry Disability Index (ODI) assessments were examined using Pearson correlation and hierarchical linear modeling.

**Results:**

A total of 93 participants were enrolled; of these, 35 (38%) completed the program (age: mean 46, SD 16 years; female: 22/35, 63%). More than half of completers finished assessments at least every 3 days and 70% (19/27) used the rehabilitation component at least once a week. Among respondents to a Web-based feedback survey, 76% (16/21) found the daily notifications helped them remember to complete their exercises, 81% (17/21) found the system easy to use, and 62% (13/21) rated their overall experience good or excellent. Baseline Your Activities of Daily Living score was a significant predictor of baseline ODI score, with ODI increasing by 0.30 units for every 1-unit increase in Your Activities of Daily Living (*P*<.001). Similarly, hierarchical linear modeling analysis indicated that Your Activities of Daily Living daily assessment scores were significant predictors of ODI scores over the course of the study (*P*=.01).

**Conclusions:**

Engagement among participants who completed the Limbr program was high, and program utility was rated positively by most respondents. Your Activities of Daily Living was significantly associated with ODI scores, supporting the validity of this novel tool. Future studies should assess the effect of Limbr on clinical outcomes, evaluate its use among a wider patient sample, and explore strategies for reducing attrition.

**Trial Registration:**

ClinicalTrials.gov NCT03040310; https://clinicaltrials.gov/ct2/show/NCT03040310 (Archived by WebCite at http://www.webcitation.org/722mEvAiv)

## Introduction

Management of chronic conditions places a considerable burden on patients, communities, and health care systems worldwide [1], but evidence indicates that symptom management in chronic disease can be significantly improved through self-management interventions [2,3]. Given that mobile phone usage in the United States has become widespread in recent years and is still on the rise [4], advancements in mobile technology can be leveraged to deliver mobile health (mHealth) apps that support patients in effective self-management of chronic conditions. In a recent US study of mHealth use among primary care patients, 55% of respondents reported having a mobile phone and 70% of these had used mHealth apps for management of health conditions [5].

Conditions for which exercise therapy has been shown to be effective, such as chronic lower back pain (CLBP) [6], stand to benefit greatly from mHealth integration because sustained adherence to exercise-based rehabilitation is vital for recovery [7-9]. The effectiveness of mobile phone-based interventions for measuring and influencing physical activity has been explored in a number of studies, and there is increasing evidence that mHealth interventions that are adaptive to user preference while supplementing standard care with disease monitoring, self-reporting, education, and promoting physical therapy adherence have the potential to improve health outcomes among those living with chronic diseases [1,10,11]. Support from a health coach has also been shown to help drive mHealth app use [10,12], and remote health coaching in the form of text messages can effectively improve self-management of symptoms and promote long-term behavior change retention [13,14], including increased compliance with physical therapy [15]. Ecological momentary assessment, or “experience sampling”—including self-report surveys and sensor-assisted reminders—are effective tools for collecting in situ user data [16,17] and can be used to enhance mHealth interventions for the self-management of CLBP.

Limbr is a compliance enhancement intervention that was developed to incorporate many of these elements by packaging self-directed rehabilitation tutorial videos, personalizable visual self-report tools, health coach support, and sensor-assisted, passive activity-level tracking into a suite of mobile phone-based apps for patients with CLBP. The Limbr program aims to promote adherence to the Back Rx exercise rehabilitation regimen [18], increase engagement in self-directed management of pain (including pain, medication, and exercise tracking), and improve self-reported outcomes for pain. One novel aspect of Limbr is its use of Your Activities of Daily Living, an image-based tool for characterizing functional status [19]. Although a recent preliminary evaluation of Your Activities of Daily Living conducted among a small number of patients with arthritis suggested promise of its utility [19], it has not yet been evaluated among a larger patient group.

Despite the existence of numerous patient-targeted mobile phone apps for pain tracking, self-management, and exercise training, the implementation of mHealth technology for chronic conditions remains an important area for further research. In particular, many currently available mobile phone apps targeted at low back pain (LBP) management are low in quality, offering content that is not based on current research and has not been reviewed or tested by health care providers [20,21]. This study was performed to (1) describe patient engagement with the Limbr program, (2) describe the patient-perceived utility of the Limbr program, and (3) assess the validity of the Your Activities of Daily Living module as a quantifier of pain and disability level among patients with CLBP.

## Methods

### Study Design

This was a single-arm trial utilizing a convenience sample of 93 adult patients who visited physiatrist Vijay Vad, MD (New York, NY, USA), from January 2016 through February 2017 and were diagnosed with discogenic back pain. Included patients were required to be English speaking and to have a diagnosis of LBP with predominantly axial symptoms, persistence of symptoms for at least 3 months, lumbar intervertebral disk pathology evident on magnetic resonance imaging, and possession of a mobile phone device (iPhone models 5S and later or Android models 2.3 and later). Patients were excluded if they had a history of trauma, a history of lumbar spine surgery or severe lumbar disk degeneration prior to the beginning of the study, or concurrent pathology that could have contributed to axial low back symptoms (eg, spondylolysis, spondylolisthesis, facet arthropathy), or if their case involved a legal claim. Informed consent was obtained from patients at the initial doctor visit (onboarding) after the nature of the study had been explained. The study is registered on ClinicalTrials.gov (identifier NCT03040310), was approved by the institutional review board of the Hospital for Special Surgery (New York, NY), and was conducted in accordance with all applicable regulations.

### Intervention

Eligible patients were enrolled in an mHealth-based 3-month physical therapy program (Limbr) and received a mobile phone app suite free of charge to monitor and manage their CLBP. The program included three daily visual self-reports to assess pain, medication/coping mechanisms, and affect; self-directed rehabilitation via Back Rx video tutorials personalized for patients with discogenic back pain; and passive measurement of activity levels. At onboarding, patients underwent baseline assessments (including a Your Activities of Daily Living full assessment and an Oswestry Disability Index [ODI] [22] assessment) and received assistance with installation and setup of the mobile phone app suite. For the duration of the program, patients received remote support from a health coach available in real time, and several patient engagement methods were utilized to improve user compliance. Elements of Limbr are described in further detail subsequently.

#### Self-Reports

The daily visual self-reports—Your Activities of Daily Living [19], Medications of Daily Living, and the Photographic Affect Meter (PAM) [23]—used an experience sampling approach to collect in situ user data for the purpose of providing tailored content to users. Rather than relying on words or numbers, these questionnaires offer a variety of photographs from which the user selects those that best describe their mood/condition.

**Figure 1:**
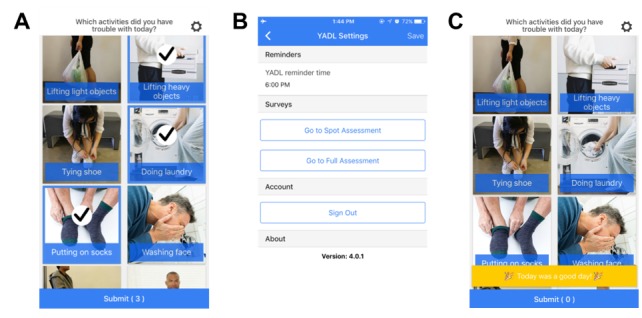
Screenshots from the Your Activities of Daily Living app: (A) daily assessment, (B) daily assessment reminders, and (C) when there was nothing to report, patients selected “Today was a good day!”.

Your Activities of Daily Living (Figure 1) [19] is an image-based survey inspired by the PAM [23] that characterizes a patient’s functional status using images representing activities of daily living (ADL) from the Western Ontario and McMaster Universities Arthritis Index [24] and Boston Activity Measure for Post-Acute Care [25], which are validated clinical measures. To complete the Your Activities of Daily Living assessment, patients used the app to select images of activities during which they recently experienced LBP-induced difficulty. The full assessment conducted at onboarding included 47 images and was intended as a substitute for a conventional, clinician-administered long-form ADL questionnaire (eg, the ODI). The Your Activities of Daily Living daily assessment was intended to provide interim reports between time points at which a long-form assessment would typically be administered and included only the images selected by the patient during the baseline full assessment.

**Figure 2:**
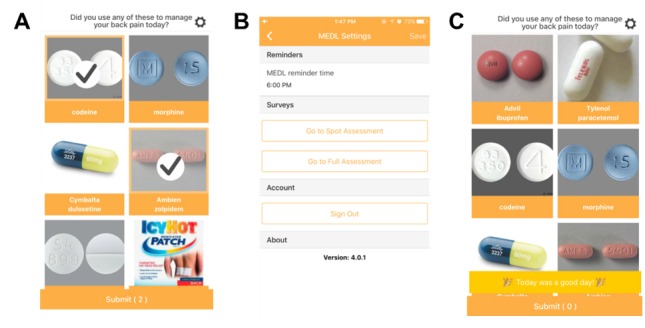
Screenshots from the Medications of Daily Living app: (A) daily assessment, (B) daily assessment reminders, and (C) when there was nothing to report, patients selected “Today was a good day!”.

Medications of Daily Living (Figure 2) is an app-based medication log with a visual interface. Similar to Your Activities of Daily Living, patients completing the daily Medications of Daily Living assessment by choosing images that characterized any LBP medication or coping strategies used over the past 24 hours. All medications and coping strategies included in Medications of Daily Living had been confirmed by the study physician as relating to LBP.

**Figure 3:**
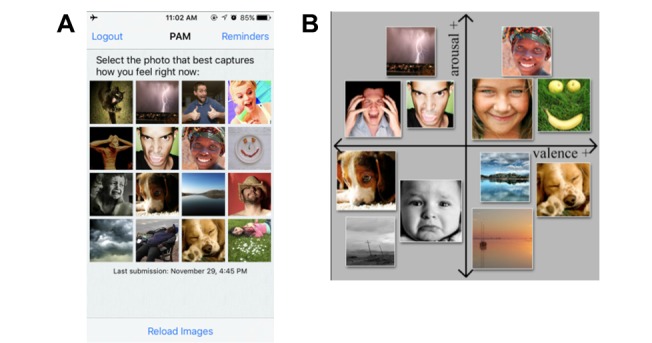
The Photographic Affect Meter (PAM) app. (A) Screenshot of the PAM visual interface, and (B) how images are arranged from low arousal and negative valence in the bottom left to high arousal and positive valence in the top right.

PAM (Figure 3), used to assess patients’ daily affect, is a rigorously validated tool for measuring emotion through a series of images [23]. Photos in the PAM are arranged in a grid from low arousal and negative valence in the bottom left, to high arousal and positive valence in the top right. To complete the daily PAM assessment, patients used the app to choose the image that best represented their emotion at the time of assessment.

#### Self-Directed Rehabilitation

The self-directed rehabilitation component of Limbr was administered via Force Therapeutics [26], an app providing a series of exercise videos tailored to patients with LBP. Patients were requested to watch three times per week. Patients used the app to view videos and to indicate if they watched the videos.

#### Activity-Level Measurement

Activity levels, including personal location and activity classification information (eg, minutes active per day, hours out of the house), were monitored via Moves [27], an app that utilizes mobile device sensors for passive collection of activity-level data.

#### Health Coach Support

Data-informed health coaching with a certified health coach was made available via Limbr Chat, a text-messaging app. (Standard iOS and Android messaging apps could not be used to maintain compliance with the Health Insurance Portability and Accountability Act of 1996 [28].) The coach in this study was familiar with the Force Therapeutics exercises and advised patients about exercise, technical issues, and personal support. The coach monitored participant data from the Limbr suite, including daily self-reports, indicators of participant compliance, and activity levels; identified trends in participants’ progress to provide personalized care; and used the Limbr Chat app to send responses and other messages, including support messages and reminders to interact with the program. The coach used casual language and abbreviations typical of text messaging (eg, “u” instead of “you”) to promote an informal relationship and reduce intimidation on the part of participants, and any patient messages were responded to within 24 hours.

#### Patient Engagement

During the study, Limbr participants were categorized according to their level of engagement with the program for the purpose of tracking and improving compliance. Patient categorization was updated three times a week on the basis of the frequency and quality of engagement with the interactive components of the system (watching videos or completing the visual self-reports). Patients were categorized as “frequently interacting” (>2 interactive components/week), “infrequently interacting” (<1 interactive component/week), and “unproductive-active” (1-2 interactive components/week).

**Figure 4:**
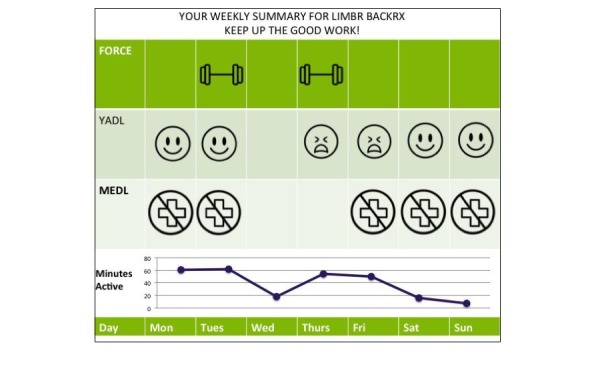
Example of a weekly summary email.

To promote sustained engagement, all frequently interacting and unproductive-active participants were sent weekly summary emails (Figure 4) consisting of a visual feedback regarding their interactions across the Your Activities of Daily Living and Medications of Daily Living visual self-reports, self-directed rehabilitation (Force Therapeutics), and passively collected mobility data. To encourage engagement among infrequently interacting participants, an email was sent reminding them to engage with the Limbr components and log their activities. Unproductive-active patients were sent personalized messages from the Limbr health coach, who worked with the patients to construct a new care plan according to individual patient needs. For example, the coach might suggest that a patient reduce interaction with the daily assessments from daily to three times per week. Finally, the Limbr health coach would check in weekly with all frequently interacting, unproductive-active, and infrequently interacting patients, inquire about their progress, and send personalized motivational messages. Examples of messages that might be sent to encourage patient engagement and/or check in regarding a patient’s progress are:

Good morning, I know it can seem like we are asking u to track a lot of things and data. Honestly, nothing is more important to your healthy outcome than the FORCE exercises. I do them myself. Please don’t forget to do them—1 set, 3 times per week AND mark them all done in the FORCE app. Thank you and good health.

I am glad the nights are getting better. Sometimes the lack of movement can stiffen the body. Series B is best thought of as the next level up shall we say on the exercises. You should keep doing the exercises you have been doing until they seem too easy, then contact Dr Vad to get his permission to move to Series B.

Patients were categorized as inactive if they had had no interaction with the interactive components for 4 successive weeks. Participants were said to have completed the program if they remained active for 12 weeks.

### Outcomes

#### Patient Engagement

Patient engagement was assessed using three outcome variables: (1) the frequency of interactions across the visual self-reports, (2) a binary outcome representing at least one viewing of the physical therapy videos versus none watched, and (3) the frequency of messages to the health coach. For outcome analysis, an interaction was defined as an instance of using Your Activities of Daily Living, Medications of Daily Living, PAM, or the Limbr Chat app during the study period. The percentage of physical therapy videos watched was automatically collected by the Force Therapeutics app. Frequency of messages was computed as the number of times a participant sent a message to the coach using the Limbr Chat app during the study period.

#### Patient-Perceived Utility of Limbr

The overall utility of the Limbr program was assessed using a Web-based feedback survey administered to all participants at the completion of the study. The feedback survey consisted of 13 questions, presented on a 5-point Likert scale, and divided into three sections that assessed the perceived helpfulness of (1) the patient engagement features (Limbr Chat app and weekly summary emails), (2) the app notifications reminding users to complete the daily self-reports and Back Rx exercises, and (3) the visual self-reports for Your Activities of Daily Living, Medications of Daily Living, and PAM. The response options for each section ranged from “strongly disagree/not useful” (5 points) to “strongly agree/very useful” (1 point).

#### Association of Your Activities of Daily Living with Conventional Pain Assessment

To determine whether the Your Activities of Daily Living visual self-report could serve as a proxy for a more traditional pain index, outcomes from the Your Activities of Daily Living assessment were compared at baseline with those from the ODI, a questionnaire that measures levels of disability in ADLs among patients rehabilitating from LBP [22]. Participants were directed to complete the ODI at onboarding (baseline) and at 2 weeks, 6 weeks, and 3 months after enrollment. The ODI was completed via Ohmage [29], a mobile survey app utilized for recording, analyzing, and visualizing participant data and administering clinical surveys.

### Statistical Analysis

Baseline patient characteristics, patient engagement, and patient-perceived utility of the Limbr program were analyzed descriptively; means and standard deviations were provided for continuous variables, and numbers and percentages were provided for discrete variables. Associations between participant characteristics and level of interaction with Limbr (total interactions and interactions per week) were analyzed using multiple linear regression to determine whether characteristics (either collectively or individually) had any effect on use of the Limbr system.

To analyze the association between Your Activities of Daily Living and ODI assessment results at baseline, Pearson correlation coefficient was calculated after confirming normality using the Shapiro-Wilk test. In addition, hierarchical linear modeling (HLM) was used to analyze the ability of Your Activities of Daily Living daily self-reports to predict ODI scores among participants who both entered multiple ODI scores and completed the full Limbr program. For the HLM analysis, the outcome was the ODI score reported on a particular day and the predictor variable was the Your Activities of Daily Living score reported closest in time to that ODI score.

## Results

### Patient Characteristics

A total of 93 participants were enrolled from January 2016 through February 2017, of which 13 dropped out after completing the onboarding session and before interacting with the Limbr components. Of the remaining 80 participants, an additional 45 dropped out before completing the 3-month study duration. This left 35 participants (age: mean 46, SD 16 years; female: 22/35, 63%; duration of symptoms: mean 19.6, SD 7.4 months; Table 1) who remained enrolled and used the Limbr interactive components throughout the study.

Patient characteristics did not have a significant collective effect on the total number of program interactions (*F*_6,27_=2.183, *P*=.08, *R*^2^=.177) or the number of program interactions per week (*F*_6,27_=2.337, *P*=.06, *R*^2^=.1956). However, age and duration of symptoms were each individual predictors of total interactions (*t*_27_=3.128, *P*=.004 and *t*_27_=–2.258, *P*=.03, respectively) and interactions per week (*t*_27_=3.159, *P*=.004 and *t*_27_=–2.355, *P*=.03, respectively; Table 2).

### Outcomes

#### Patient Engagement

**Figure 5:**
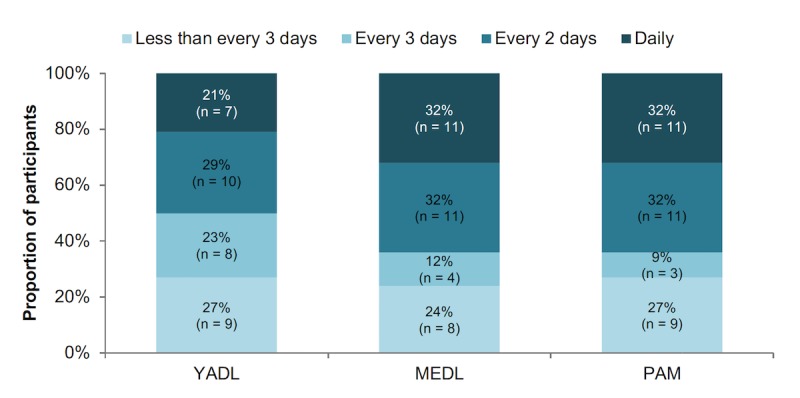
Median interaction frequency across daily self-reports for Your Activities of Daily Living (YADL), Medications of Daily Living (MEDL), and the Photographic Affect Meter (PAM).

**Figure 6:**
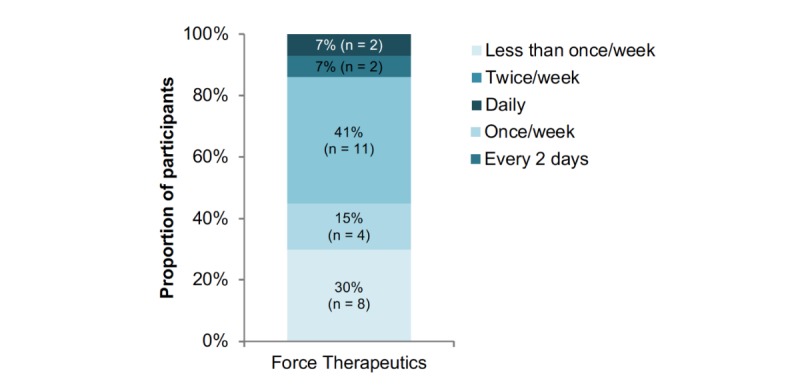
Median interaction frequency for daily self-reports for Force Therapeutics. Note there were different frequencies of data reported from Force Therapeutics versus the other assessments.

The 35 participants who completed the program averaged 96 total interactions with the three daily self-reports over the 12-week study, roughly evenly distributed among Your Activities of Daily Living, Medications of Daily Living, and PAM. The number of interactions per week ranged from 1 to 29, with a mean of 8 (SD 7). On average, participants who interacted with the daily assessments between daily and every 3 days comprised slightly over 50% of the study group. Median participant interaction frequency across assessments is depicted in Figure 5. Participants were instructed to watch the Force Therapeutics instructional videos only 1 to 3 times a week, as opposed to daily. Interaction data shows that 70% (19/27) of participants interacted with Force Therapeutics a median of at least once a week (Figure 6).

**Table 1:** Patient characteristics (N=35).

Characteristic		Completing participants
Age (years), mean (SD)		46 (16)
Female, n (%)		22 (63)
Body mass index, mean (SD)		25.4 (4.0)
Duration of symptoms (months), mean (SD)		19.6 (7.4)
**Mobile phone operating system, n (%)**		
	Android	4 (11)
	iOS	31 (89)

**Table 2:** Associations between participant characteristics and program interaction measures (N=35).

Variable	Total interactions^a^		Interactions per week^a^	
	*t*_27_	*P* value	*t*_27_	*P* value
Age, years	3.128	.004	3.159	.004
Male sex	–1.167	.25	–0.997	.33
Body mass index	0.509	.63	0.702	.49
Duration of symptoms	–2.258	.03	–2.355	.03
iOS mobile phone operating system	1.197	.24	1.279	.21

^a^An interaction was defined as an instance of using Your Activities of Daily Living, Medications of Daily Living, PAM, or the Limbr Chat app during the study period.

**Figure 7:**
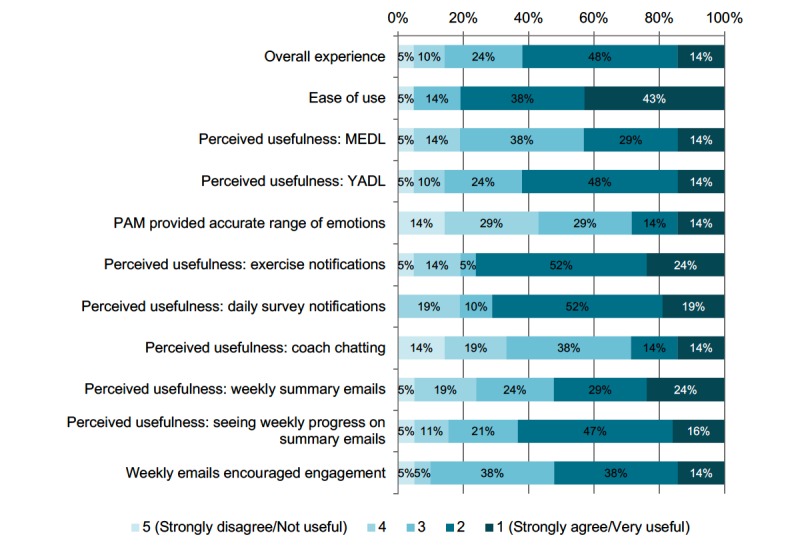
Feedback survey results. Surveys were scored on a 5-point Likert scale with response options ranging from “strongly disagree/not useful” (5 points) to “strongly agree/very useful” (1 point). Percentages may not sum to 100% because of rounding. YADL: Your Activities of Daily Living; MEDL: Medications of Daily Living; PAM: Photographic Affect Meter.

A total of 147 messages were sent from participants to the coach using the Limbr Chat app. The majority of these (73/147, 49.7%) were tech support messages (eg, “I cannot seem to log in to force. Can you help?”), with the next most common type (47/147, 32.0%) comprising messages about the Force Therapeutics exercises (eg, “Yes my knee reaches. Not the hip flexor. The hip flexor is tight and I feel pain. So was wondering if that’s normal and if not then I shouldn’t stretch that much.”). A smaller percentage (23/147, 15.6%) were medical messages (eg, “Thank you. I have had extreme back pain for the last 2 days. I hurts to sit, sleep, and bend at the moment”), and 2.7% of messages (4/147) recorded participant criticisms of the system.

#### Patient-Perceived Utility of Limbr

Feedback surveys were returned by 21 participants; a question-by-question breakdown of participant responses in presented in Figure 7. Among respondents, 11 of 21 (52%) found the daily self-reports to be helpful in tracking pain-related ADL functionality, medication use, and affect. In particular, 13 of 21 (62%) found that the Your Activities of Daily Living daily assessment helped them track the activities of daily living affected by their back pain; 16 of 21 (76%) and 15 of 21 (71%) agreed that the daily notifications were helpful in reminding them to complete the Force Therapeutics exercises and daily surveys, respectively; 17 of 21 (81%) found the Limbr system easy to use; and 13 of 21 (62%) rated their overall experience as either good or excellent.

#### Association of Your Activities of Daily Living With Conventional Pain Assessment

Baseline Your Activities of Daily Living and ODI scores were found to be significantly associated (Pearson correlation coefficient=.551, *P*<.001). Linear regression modeling further revealed that the baseline Your Activities of Daily Living score was a significant predictor of baseline ODI score, with ODI increasing by 0.30 units for every 1-unit increase in Your Activities of Daily Living (*P*<.001). Similarly, HLM analysis (among the 14 patients with multiple ODI scores who completed the full Limbr program) indicated that Your Activities of Daily Living daily assessment scores were significant predictors of ODI scores recorded over the course of the study, with ODI increasing by 0.33 units for every 1-unit increase in Your Activities of Daily Living (*P*=.01).

## Discussion

In this pilot study conducted among patients with CLBP, engagement with the Limbr compliance enhancement intervention was high among those who finished the program; the majority of completers interacted with the daily self-reports multiple times per week and 70% used the self-directed rehabilitation component as directed. Approximately half of feedback survey respondents found the daily self-report components of Limbr to be helpful, and more than 70% indicated that the daily notifications helped them remember to perform their rehabilitation exercises. Moreover, the Your Activities of Daily Living assessment was found to be significantly associated with conventional pain assessment scores, thereby validating its utility as a novel quantifier of pain and disability level. These findings suggest that Limbr has substantial potential as an approach to promoting patient engagement and self-directed rehabilitation adherence for CLBP management.

Although US data regarding mHealth interventions for patients with CLBP are limited, evidence exists that Web- or mobile-based strategies can be effective for reducing pain and improving self-management in this population [30-32]. In this study, participants who completed the Limbr program exhibited a high level of engagement throughout the trial, frequently interacting with the self-report modules as well as with Force Therapeutics. It is particularly encouraging that most respondents found Limbr helpful in remembering to engage with self-directed rehabilitation, as rehabilitation adherence is critical for maximum improvement in physical function among patients with CLBP [7-9]. In addition, the sustained use of Your Activities of Daily Living, Medications of Daily Living, and PAM observed throughout the trial suggests that patients may find these visual assessments—which provide a simple and intuitive interface, can be completed quickly, and are readily adapted to mobile devices—to be easier to use than conventional reporting methods such as text-based surveys [19,23].

The significant association of the Your Activities of Daily Living assessment with the ODI scores is a key finding of this study. As a visual survey, Your Activities of Daily Living leverages the inherent ambiguities of images to mitigate some of the limitations of conventional pain assessments [19]. For example, although it is not possible for a standardized survey to contain a comprehensive list of every ADL that could be relevant for every patient, the images in Your Activities of Daily Living are open to individual interpretation and, therefore, can be used by different patients to express a wider range of ADL experiences [19]. Furthermore, standard ADL assessments are typically performed in an office setting in association with a clinical encounter, limiting their ability to reflect day-to-day variability in ADL performance. In contrast, Your Activities of Daily Living is mobile-friendly and can be completed by patients at any time without assistance, greatly increasing the scope and granularity of the ADL information that can be captured. Validation of Your Activities of Daily Living opens the door to the creation of mHealth apps that are capable of reliably measuring patient-reported pain outcomes on a day-to-day basis. The relationship between Your Activities of Daily Living daily assessments and pain-related disability as assessed by the ODI should be explored in further studies.

Participant attrition in this study was high; of 93 patients enrolled, only 35 (38%) completed the 3-month program. Although this level of attrition is substantial, it is not unusual among mHealth interventions, for which the challenge of many participants discontinuing the intervention and/or being lost to follow-up is widely acknowledged [33]. For example, only 32 of 180 (18%) study enrollees were retained after 12 weeks in one recent analysis of a multidisciplinary LBP pain treatment app, despite the fact that those who completed the program experienced significant reductions in pain [34]. Similarly, only 25% of participants in a 1-year internet-mediated exercise intervention for patients with CLBP maintained at least 80% compliance with required data uploads for the duration of the study [32]. Excellent participant retention was observed in the 4-month FitBack randomized controlled trial, which demonstrated greater pain reduction among program users versus those in comparison groups. Of 597 initial enrollees, 580 (97%) submitted assessments at all three designated time points [30]. It is noteworthy, however, that FitBack participants received cash rewards for submitting assessments, and the degree to which those who submitted all three assessments engaged with the intervention program on a daily or weekly basis (eg, tracking pain and pain-management activities or watching instructional videos) is unknown [30]. Furthermore, FitBack is a comparatively simple Web-based program in contrast to Limbr, which required installation, maintenance, and utilization of seven separate component apps. The complexity of the combined interactions and maintenance tasks required of Limbr participants may have been a factor in the high dropout rate. As nearly half of the chat messages sent by participants in this study were categorized as tech support-related, the possibility that technical difficulties contributed to attrition also cannot be discounted.

Considerable effort was made to improve patient engagement and reduce attrition during the course of this study. First, patient input from feedback surveys and calls was used to improve usability and enhance the user experience of the daily self-reports; for instance, two of the reports (Your Activities of Daily Living and Medications of Daily Living) were updated and moved from beta testing to the app store. Before this change was made, beta testing expirations frequently required patients to manually update their apps, and there was a significant difference in device usage between iOS and Android phones. After the update, however, there was no difference in usage between operating systems (data not shown). This example highlights the importance of making the patient experience as seamless as possible to promote engagement with the intervention.

The other major effort to enhance patient interaction comprised personalized Limbr Chat messages from the health coach, which were tailored according to participant data collected via the Limbr suite. A post hoc analysis revealed that 408 health coach messages were sent, with the largest proportion categorized as engagement (169/408, 41.4%), followed by technical support (113/408, 27.6%), and medical/exercise-related messages (60/408, 14.7%). Findings from previous studies suggest that interactions with a health coach, including two-way messaging systems similar to that used in Limbr [35], tend to increase patient engagement with the intervention [35] and promote improved self-management of chronic pain conditions [36]. However, of the messages sent from patients to the coach, only 74 of 147 were unrelated to technical support. Although we expected more patient messaging, Limbr did little to encourage patients to engage in a two-way exchange or spontaneously send messages to the coach. Messages from the coach rarely prompted the patient to respond, as the coach was not instructed to do so, and the system itself did not actively guide the patient to the chat app unless there was a new message waiting. The low number of patient-sent messages is not necessarily an indicator of poor engagement, because patients who were highly engaged in the exercising and reporting may have felt little need for communication with the coach. Nevertheless, the effectiveness of various types of health coach messaging is an important area for future research.

Despite the relatively high attrition rate, the overall utility of the Limbr system was scored positively by the majority of respondents and some individual components were widely found to be useful; for example, the weekly summary emails were met with optimum positive feedback. On the other hand, reception of other Limbr components varied considerably across participants. Daily notifications and recurrent coach messages were particularly polarizing, seen as vexatious by some participants but highly motivating by others. This finding underscores the need for mHealth interventions to take a personalized approach to engagement rather than relying on a single method of promoting compliance. Additional studies to characterize which engagement efforts are most effective both overall and for particular patient populations could help enable the design of highly personalizable interventions in the future. Other changes that could reduce attrition and enhance engagement in a future iteration of the Limbr system include combining the disparate components into a single app designed with user-first principles and fewer technical barriers, conducting more formal user testing and employing analytical techniques such as conversion funnel optimization prior to considering a larger trial and emphasizing two-way messaging between patient and coach.

Our results should be considered in light of several limitations. Because the trial was conducted among a small convenience sample of patients with CLBP, the applicability of the study findings to other patient populations is unknown and should be assessed in future studies. In addition, because there was no comparison group, the study outcomes cannot be definitively attributed to use of the Limbr suite. Finally, although the reasons underlying the high dropout rate of the trial warrant further exploration, this study was not designed to analyze the causes of patient attrition or the types of engagement efforts that were most helpful in promoting compliance with the intervention.

The findings of this pilot study suggest that the Limbr program shows promise as an approach to enhancing patient self-management and adherence to self-directed rehabilitation for CLBP. Engagement among participants who completed the program was high, and the utility of the program was rated positively by the majority of respondents. Our results also support the validity of the Your Activities of Daily Living visual self-assessment for quantifying pain and disability level. Future studies should assess the effect of Limbr on clinical outcomes, evaluate its use among a wider patient sample, and explore strategies for reducing attrition.

## Abbreviations

ADL: activities of daily living
CLBP: chronic low back pain
HLM: hierarchical linear modeling
LBP: low back pain
mHealth: mobile health
ODI: Oswestry Disability Index
PAM: Photographic Affect Meter

## References

[1] Whitehead L, Seaton P (2016). The effectiveness of self-management mobile phone and tablet apps in long-term condition management: a systematic review. J Med Internet Res.

[2] Coleman MT, Newton KS (2005). Supporting self-management in patients with chronic illness. Am Fam Physician.

[3] Turner A, Anderson JK, Wallace LM, Bourne C (2015). An evaluation of a self-management program for patients with long-term conditions. Patient Educ Couns.

[4] (2018). Pew Research Center.

[5] Bauer AM, Rue T, Keppel GA, Cole AM, Baldwin L, Katon W (2014). Use of mobile health (mHealth) tools by primary care patients in the WWAMI region Practice and Research Network (WPRN). J Am Board Fam Med.

[6] Foster NE, Anema JR, Cherkin D, Chou R, Cohen SP, Gross DP, Ferreira PH, Fritz JM, Koes BW, Peul W, Turner JA, Maher CG, Lancet Low Back Pain Series Working Group (2018). Prevention and treatment of low back pain: evidence, challenges, and promising directions. Lancet.

[7] Mannion AF, Helbling D, Pulkovski N, Sprott H (2009). Spinal segmental stabilisation exercises for chronic low back pain: programme adherence and its influence on clinical outcome. Eur Spine J.

[8] (2003). World Health Organization.

[9] Cecchi F, Pasquini G, Paperini A, Boni R, Castagnoli C, Pistritto S, Macchi C (2014). Predictors of response to exercise therapy for chronic low back pain: result of a prospective study with one year follow-up. Eur J Phys Rehabil Med.

[10] Bort-Roig J, Gilson ND, Puig-Ribera A, Contreras RS, Trost SG (2014). Measuring and influencing physical activity with smartphone technology: a systematic review. Sports Med.

[11] Krishna S, Boren SA, Balas EA (2009). Healthcare via cell phones: a systematic review. Telemed J E Health.

[12] Alley S, Jennings C, Plotnikoff RC, Vandelanotte C (2016). Web-based video-coaching to assist an automated computer-tailored physical activity intervention for inactive adults: a randomized controlled trial. J Med Internet Res.

[13] Job JR, Spark LC, Fjeldsoe BS, Eakin EG, Reeves MM (2017). Women's perceptions of participation in an extended contact text message-based weight loss intervention: an explorative study. JMIR Mhealth Uhealth.

[14] Spark LC, Fjeldsoe BS, Eakin EG, Reeves MM (2015). Efficacy of a text message-delivered extended contact intervention on maintenance of weight loss, physical activity, and dietary behavior change. JMIR Mhealth Uhealth.

[15] Lilje SC, Olander E, Berglund J, Skillgate E, Anderberg P (2017). Experiences of older adults with mobile phone text messaging as reminders of home exercises after specialized manual therapy for recurrent low back pain: a qualitative study. JMIR Mhealth Uhealth.

[16] Stone AA, Shiffman S (2002). Capturing momentary, self-report data: a proposal for reporting guidelines. Ann Behav Med.

[17] Froehlich J, Chen M, Consolvo S, Harrison B, Landay J (2007). MyExperience: a system for in situ tracing and capturing of user feedack on mobile phones.

[18] Vad VB, Bhat AL, Tarabichi Y (2007). The role of the Back Rx exercise program in diskogenic low back pain: a prospective randomized trial. Arch Phys Med Rehabil.

[19] Yang L, Freed D, Wu A, Wu J, Pollak J, Estrin D (2016). Your Activities of Daily Living (YADL): An image-based survey technique for patients with arthritis.

[20] Lalloo C, Jibb LA, Rivera J, Agarwal A, Stinson JN (2015). “There's a pain app for that”: review of patient-targeted smartphone applications for pain management. Clin J Pain.

[21] Machado GC, Pinheiro MB, Lee H, Ahmed OH, Hendrick P, Williams C, Kamper SJ (2016). Smartphone apps for the self-management of low back pain: a systematic review. Best Pract Res Clin Rheumatol.

[22] Fairbank JC, Pynsent PB (2000). The Oswestry Disability Index. Spine (Phila Pa 1976).

[23] Pollak J, Adams P, Gay G (2011). PAM: a photographic affect meter for frequent, in situ measurement of affect.

[24] McConnell S, Kolopack P, Davis AM (2001). The Western Ontario and McMaster Universities Osteoarthritis Index (WOMAC): a review of its utility and measurement properties. Arthritis Rheum.

[25] Jette DU, Stilphen M, Ranganathan VK, Passek S, Frost FS, Jette AM (2015). Interrater Reliability of AM-PAC “6-Clicks” Basic Mobility and Daily Activity Short Forms. Phys Ther.

[26] (2017). Force Therapeutics.

[27] (2017). ProtoGeo Oy.

[28] (2013). HHS.gov.

[29] Ramanathan N, Alquaddoomi F, Falaki H, George D, Hsieh C, Jenkins J, Ketcham C, Longstaff B, Ooms J, Selsky J, Tangmunarunkit H, Estrin D (2012). Ohmage: an open mobile system for activity and experience sampling.

[30] Irvine AB, Russell H, Manocchia M, Mino DE, Cox GT, Morgan R, Gau JM, Birney AJ, Ary DV (2015). Mobile-Web app to self-manage low back pain: randomized controlled trial. J Med Internet Res.

[31] Chiauzzi E, Pujol LA, Wood M, Bond K, Black R, Yiu E, Zacharoff K (2010). painACTION-back pain: a self-management website for people with chronic back pain. Pain Med.

[32] Krein SL, Kadri R, Hughes M, Kerr EA, Piette JD, Holleman R, Kim HM, Richardson CR (2013). Pedometer-based internet-mediated intervention for adults with chronic low back pain: randomized controlled trial. J Med Internet Res.

[33] Eysenbach G (2005). The law of attrition. J Med Internet Res.

[34] Huber S, Priebe JA, Baumann K, Plidschun A, Schiessl C, Tölle TR (2017). Treatment of low back pain with a digital multidisciplinary pain treatment app: short-term results. JMIR Rehabil Assist Technol.

[35] Jamison RN, Jurcik DC, Edwards RR, Huang C, Ross EL (2017). A pilot comparison of a smartphone app with or without 2-way messaging among chronic pain patients: who benefits from a pain app?. Clin J Pain.

[36] Allen KD, Oddone EZ, Coffman CJ, Datta SK, Juntilla KA, Lindquist JH, Walker TA, Weinberger M, Bosworth HB (2010). Telephone-based self-management of osteoarthritis: a randomized trial. Ann Intern Med.

